# Pathologies and technologies for digital therapeutics: An analysis of global clinical trials

**DOI:** 10.1371/journal.pdig.0001750

**Published:** 2026-10-14

**Authors:** Paolo Rocco, Valentina Pagella, Umberto M. Musazzi, Sara Manellari, Elena Paola Lanati, Paola Minghetti

**Affiliations:** 1 Department of Pharmaceutical Sciences, University of Milan, Milan, Italy; 2 INDICON S.r.l. Società Benefit, Milan, Italy; The University of Sheffield, UNITED KINGDOM OF GREAT BRITAIN AND NORTHERN IRELAND

## Abstract

CDigital therapeutics (DTx) are software-based medical devices that deliver evidence-based therapeutic interventions directly to patients. While the clinical use of DTx is expanding rapidly, regulatory fragmentation and the absence of harmonized legal definitions hinder their assessment and reimbursement policies, as well as their integration into healthcare systems. There is currently a lack of comprehensive overviews of DTx clinical development, which are essential for informing policy, research and regulatory priorities. This paper provides an updated overview of randomised clinical trials involving DTx. 254 eligible trials on DTx, (defined according to ISO/TR 11147:2023) registered through January 2025 were identified through a search on C*linicalTrials.gov*. Mobile applications represent the most prevalent technology (65%), followed by web-based tools and virtual reality (20%). Psychiatric disorders are the primary therapeutic target (52% of the studies) with insomnia, depression, and anxiety being the most addressed conditions. Trials were primarily conducted in North America (41%) and Europe (32%), where Germany, Spain, and Italy represent the most active countries. The analysis of clinical trials registrations on ClinicalTrials.gov reveals a heterogenous geographical distribution of DTx development but also a clear direction in the type of conditions for which they are mostly indicated, namely psychiatric and nervous system disorders. The fragmented policies on DTx reimbursement, even among EU countries, and the different capacity of governments to fund DTx research and development emerge as key factors, along with cultural and ethical issues, in determining the willingness of the industry to invest in these devices.

## Author summary

When a software is intended by its manufacturer to treat, prevent, diagnose or monitor a pathologic condition, it cannot be considered as a general use product. Indeed, in most legislative framework, it is a Medical Device and, as such, it is subject to strict legislation which is in place to guarantee its safety and efficacy. Nomenclature, market access and reimbursement policies for such products vary worldwide, but there is wide acceptance of the term “Digital Therapeutics” (DTx) to mean software with a therapeutic function, as formalized by the ISO. In the paper we investigate, through the analysis of DTx clinical trials registrations in ClinicalTrials.gov, the regional distribution of active studies, the type of technology most actively researched (mobile app, web-based app, virtual reality or other) and the therapeutic indications. Our analysis highlighted on the one hand a heterogenous worldwide distribution of DTx, which reflects a fragmented landscape of reimbursement policies and funding capacity. On the other hand, a clear direction in both the type of technology employed (mobile apps) and in the prevalent therapeutic indications (psychiatric and nervous system disorders) clearly emerged.

## 1. Introduction

Software with a medical purpose brings the promise of a revolution in patient care and management, through advanced connectivity and data analytics. It represents a promising facet of modern healthcare, harnessing the power of software and Artificial Intelligence to deliver diagnosis, monitoring or evidence-based interventions.

Among medical software, Digital therapeutics (DTx) hold a position of prominence as they are intended to be used directly by patients and require evidence of efficacy. As a new therapeutic approach based on digital technology, the commercialization and clinical application of Digital Therapeutics (DTx) are rapidly increasing, with growing demand for their expansion into new clinical areas [1].

According to the ISO/TR 11147:2023 definition, DTx are “health software intended to treat or alleviate a disease, disorder, condition, or injury by generating and delivering a medical intervention that has a demonstrable positive therapeutic impact on a patient’s health” [2]. A binding definition is still lacking, but the medical purpose of these products qualifies them as Medical Devices in most countries worldwide. As such, they are subject to strict regulations, such as the Medical Devices Regulation (MDR) in the European Union (EU) [3], where software intended for medical purposes - such as treating, curing, preventing disease, or supporting medical diagnosis - is called medical device software (MDSW) [4]. The same category goes under other names in other jurisdiction, such as Software as a Medical Device (SaMD)/ Software in a Medical Device (SiMD), e.g., in the USA, Japan, South Korea [5].

However, the class of MD or MDSW remains too broad to be perceived by consumers and manufacturers as a clearly identifiable category of marketed products, as it includes software intended for both healthcare professionals and patients. That is why regulatory harmonization on the definition of these technologies, not only DTx, but any MDSW not embedded in a hardware medical device and intended to be used by a layperson, alone or assisted by a healthcare professional, for a specific medical purpose. These products could be defined as a patient-managed Digital Medical Devices (pDMDs), of which DTx represent the subgroup with a therapeutic purpose. The classification and nomenclature of pDMDs, including DTx, have been treated extensively in a previous paper [6].

Currently, DTx are either available on the market or under development for a wide range of medical conditions, including diabetes, oncology treatment management, and neuropsychiatric disorders such as anxiety, depression, and substance use disorder [7]. Although DTx have specific characteristics - most notably, their therapeutic use by non-healthcare professionals - most public and regulatory authorities do not assign them a distinct regulatory category from other MDs or even medical device software [3].

In addition, market access and reimbursement policies, along with financial support for efficacy studies, vary consistently between regions and countries worldwide, but prove crucial in encouraging manufacturers to accept the challenge of researching and marketing DTx. In Europe, Germany has been the first country, since 2020, to provide a reimbursement mechanism through public insurance coverage (which covers about 90% of the population) and fast track for DTx with DiGA (*Digitale Gesundheitsanwendungen*, Digital Health Applications) and selective contracts. To qualify as DiGA, a medical device software must be Class I or IIa, intended to be used only by the patient (or by the patient and the healthcare provider) and have clinical evidence gathered in at least one retrospective comparative study. France introduced a reimbursement and fast-track mechanisms for DTx and other pDMD, such as remote patient monitoring products (RPM), since 2023, through the PECAN (*Prise En Charge Anticipée Numérique*) pathway (one year non-renewable reimbursement) or through the *Droit Commun* pathway for technologies demonstrating sufficient clinical value. In Germany and France, the legislator has also provided a 12-month conditional reimbursement supporting completion of studies to generate clinical evidence [8]. Other European countries have not been offering so far for DTx market access pathways leading to reimbursement, although in some of them, such as the UK and Italy, initiatives aimed to assess those technologies in view of reimbursement are being discussed. In the USA, the situation is heterogenous, starting with no coverage by Medicare to partial coverage by Medicare Advance to full coverage for Class II and Class III MDs by private insurance companies.

This study aims to provide an overview of the state-of-the-art in digital therapeutics through a comprehensive screening of DTx clinical trials globally conducted, in order to highlight prevalent therapeutic areas, leading countries, and types of devices developed or in development.

## 2. Materials and methods

### 2.1. Inclusion/exclusion criteria

Since a binding legal definition of DTx is still lacking in the EU, the identification of DTx in this work has been based on both the ISO/TR 11147:2023 definition^*2*^ and the Digital Therapeutics Alliance’s DTx evaluation process [9].

DTx meeting the following criteria have been included in the analysis:

- software with a medical purpose, i.e., treating or alleviating a disease, disorder, condition, or injury or employed in secondary or tertiary prevention;
- software intended to be used by the patient, alone or with the support of healthcare providers;
- software, the medical purpose of which, is achieved through the main digital function (the physician can have a supportive role);
- software that is stand-alone or combined with other components to form a system (a combination of interacting elements organised to achieve one or more purposes), functioning through general purpose hardware (smartphones, tablets, computers, watches, headphones) or input/output devices (wearables, sensors).

The following products were excluded from the study:

- software without a medical purpose, which include software app for general wellbeing or remote monitoring products;
- software for primary prevention or to promote wellness and a healthy lifestyle;
- software that exclusively supports data recognition or diagnosis (remote monitoring products);
- software designed solely to support communication or exchange of medical referrals between healthcare professionals and patients;
- healthcare professionals-facing software; software designed to assist HCPs in diagnosing, monitoring or managing patients’ data;
- software integrated with a specific hardware medical device, to be used only in that combination.

### 2.2. Methodology and source analysis

A search was conducted on clinicaltrials.gov [10] to identify clinical trials investigating DTx, with the aim of creating a database updated as of January 2025. The following key words were entered into the field: “Intervention/Treatment”: “Digital Therapeutics”, “Digital Therapeutic”, “Digital Therapy”, “Digital Therapies”. Initially, 1888 clinical trials were identified and transferred to an Excel spreadsheet to select and compare the data of interest. The selection was limited to studies classified under the “Study Type” and “Study Status” categories as “Interventional”, “Randomized”, or “Not Applicable (NA)”. Pilot studies were also included. Non-randomised studies as well as those marked as “Terminated” or “Withdrawn”, i.e., studies terminated earlier than planned or before the start of the trial, were excluded (Fig 1). This choice was informed by existing literature, which shows that approved Digital Therapeutics (DTx) have been evaluated mostly through randomized clinical trials (RCTs) [1]. Following these criteria, 1290 clinical trials remained. The data sheets of these trials were carefully examined and further screened according to the previously defined selection criteria. If the information was not sufficient to qualify the device as a digital therapy, a more in-depth investigation was conducted by consulting the manufacturer’s website. This way, 254 clinical studies fulfilling the selection criteria were identified.

**Fig 1:**
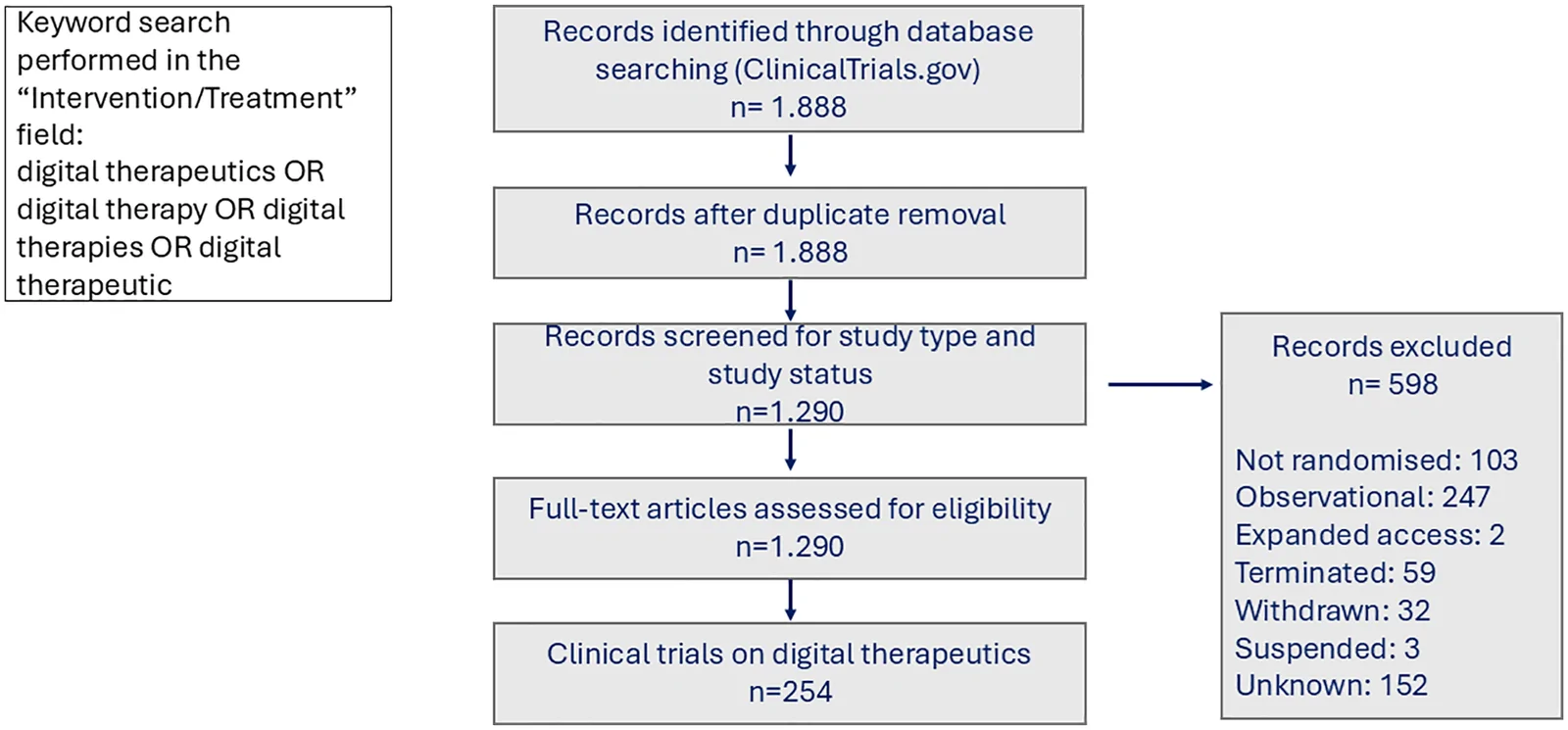
Study selection flow diagram.

The resulting dataset (S2 Dataset) served as the foundation for further analyses concerning the type of technology developed, the therapeutic areas addressed and the countries leading DTx development (the country where the trial was conducted). The countries in which the clinical studies were conducted were grouped by continent (further details can be found in S1 Appendix). The selected studies were classified according to the therapeutic area targeted and grouped into the following macro areas according to Medical Dictionary for Regulatory Activities Terminology (MedDRA), SOC level: immune system disorders; c*ardiac disorders; ear and labyrinth disorders; gastrointestinal disorders; general disorders and administration site conditions; Injury, poisoning and procedural complications; metabolism and nutrition disorders; musculoskeletal and connective tissue disorders; neoplasms benign, malignant and unspecified; nervous system disorders; psychiatric disorders; renal and urinary disorders; reproductive system and breast disorders; respiratory, thoracic and mediastinal disorders.*

The list of clinical trials collected was used as the starting point for further analyses such as: type of device, therapeutic area and geographical distribution.

A limitation of the study stems from basing the analysis solely on ClinicalTrials.gov, which may have caused an underestimation of European and Asian trials included only in regional registries.

## 3. Results

### 3.1. Device technology

The technological landscape of DTx was examined to categorize the main types of digital platforms employed across the identified clinical trials. Five categories were identified: mobile applications (apps), web apps, video games, virtual reality (VR), and apps used in combination with wearable devices. Here, a mobile app is defined as an application developed specifically to be used on mobile devices. However, applications accessible via both the web and mobile devices were also included in this class.

Mobile applications (65.35%, n = 166) stood out as the prevailing technology among the identified trials (Fig 2), a remarkable growth of 155%, compared to data from a similar study run in 2021, which reported 65 studies employing an app as their technological platform. ^7^

**Fig 2:**
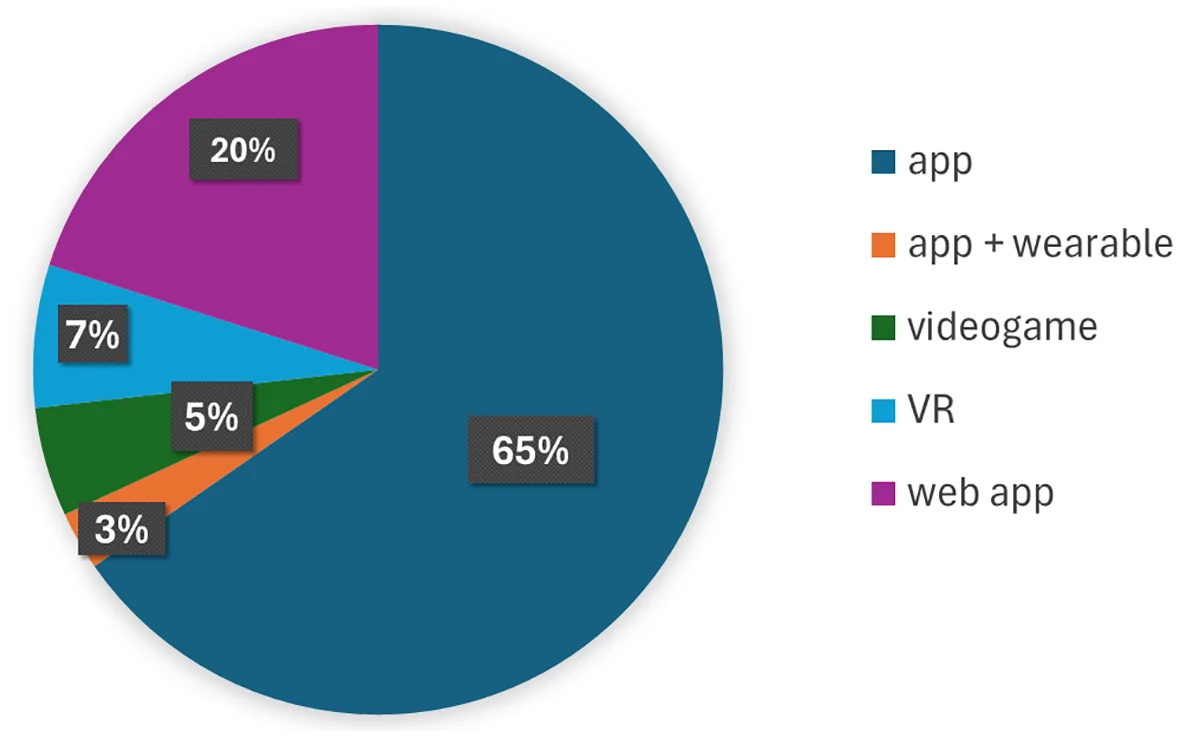
Analysis of technological devices.

In addition to this main category, web apps account for 20.08% (n = 51) of technological devices, followed by virtual reality 6.69% (n = 17). Finally, videogames and apps associated with wearable devices or sensors are less common, accounting for 5.12% (n = 13) and 2.76% (n = 7), respectively.

### 3.2. Therapeutic area

The analysis of the therapeutic areas addressed by digital therapeutics (DTx) in the reviewed clinical studies revealed a clear predominance of treatments of psychiatric disorders, which accounted for 51.57% of the total (n = 131). Of these, insomnia, depression and anxiety constitute the most prevalent pathologies (Table 1). The high number of DTx for anxiety and depression can be attributed to their increasing global prevalence [11], further exacerbated by the COVID-19 pandemic [12], and the well-established efficacy of cognitive behavioural therapy (CBT), a common component of DTx [13]. CBT is also frequently used and effective in the treatment of opioid use disorder (OUD), where it supports behaviour change and relapse prevention [14]. Additionally, in disorders such as bipolar disorder, personalized approaches — e.g., online psychoeducation and tailored digital interventions — have shown greater effectiveness compared to standard treatments [15].

**Table 1:** Analysis of therapeutic area.

Therapeutic area	Nr. clinical trials	Therapeutic area	Nr. clinical trials
**Psychiatric disorders**	**131**	**Nervous system disorders**	**28**
Insomnia	30	Multiple Sclerosis	6
Depression	26	Cognitive Impairment	4
Anxiety	14	Cognitive Decline and Dementia	4
ADHD	12	Migraine	3
Opioid use disorders	12	Parkinson Disease	3
Schizophrenia	9	Visual fields Defects	3
Behavioural disorders	7	Developmental Disability	1
Psychosis	5	Dysarthria	1
Eating Disorders	4	Amyotrophic Lateral Sclerosis (ALS)	1
Post traumatic stress disorders (PTSD)	4	Stuttering	1
Alcohol Abuse	2	Tic Disorder	1
Cognitive Deficits	2		
Methamphetamine Abuse	2		
Hallucinations	1		
Substance Use Disorders	1		
**Neoplasms benign, malignant and unspecified**	**14**	**Metabolism and nutrition disorders**	**14**
Cancer	13	Diabetes	7
Melanoma	1	Obesity	6
		Hypercholesterolemia/ Dyslipidemias	1
**Cardiac disorders**	**11**	**General disorders and administration site conditions**	**11**
Stroke	6	Chronic pain	10
Hypertension	3	Back Pain	1
Acute Myocardial Infarction	1		
Coronary Artery Disease	1		
**Musculoskeletal and connective tissue disorders**	**10**	**Respiratory, thoracic and mediastinal disorders**	**9**
Fibromyalgia	4	COPD	3
Arthritis	3	Asthma	2
Patellofemoral Pain Syndrome	2	Long COVID	1
Post Partum Lumbopelvic Pain	1	Lung Diseases, Interstitial/ Pulmonary Fibrosis	1
		Post COVID-19 Condition	1
		Rhinitis	1
**Renal and urinary disorders**	**6**	**Gastrointestinal disorders**	**6**
Urinary Incontinence	2	Irritable Bowel Syndrome (IBS)	6
Chronic Kidney Diseases	1		
Kidney Disease	1		
Overactive Bladder	1		
Stress Urinary Incontinence	1		
**Reproductive system and breast disorders**	**5**	**Injury, poisoning and procedural complications**	**5**
Endometriosis	2	Orthopedic Trauma	5
Dysmenorrhea	1	**Hepatobiliary disorders**	**2**
Female Sexual Dysfunction	1	Cirrhosis	1
Polycystic ovary syndrome (PCOS)	1	Liver Diseases	1
**Immune system disorders**	**1**	**Ear and labyrinth disorders**	**1**
Psoriasis	1	Hearing disorders	1
**Total nr. clinical trials**	**254**		

Psychiatric disorders were followed, at a significantly lower share, by nervous system disorders (11.02%; n = 28) where cognitive rehabilitation via digital devices is increasingly recognized as a promising approach to enhance brain neuroplasticity^9^. Neoplasms (benign, malignant, or unspecified) accounted for 5.51% (n = 14), the same for metabolism and nutrition disorders (5.51%; n = 14) (Table 1). In oncology, digital tools support care by enabling the collection of electronic patient-reported outcomes (ePROs) and remote monitoring, leading to better symptom management, reduced symptom distress, fewer unplanned hospitalizations, and ultimately, improved quality of life and survival [16]. In the context of metabolic diseases, digital therapeutics have become an established tool for lifestyle intervention in the management of diabetes. These technologies empower patients to take greater control of their condition through self-management, while also providing clinical decision support that benefits both patients and healthcare providers [17].

Studies related to other technologies were present in less significant quantity: general disorders and administration site conditions (4.33%; n = 11), cardiac disorders (4.33%; n = 11), musculoskeletal and connective tissue disorders (3.94%; n = 10). Notably, within neurology, the use of tablets, smartphones, and virtual reality software has enabled more effective training by providing enhanced sensory stimulation^9^. Additional categories included respiratory, thoracic, and mediastinal disorders (4.64%; n = 9), renal and urinary disorders (2.36%; n = 6), gastrointestinal disorders (2.36%; n = 6), reproductive system and breast disorders (1.97%; n = 5), and injury, poisoning, and procedural complications (1.97%; n = 5). Finally, the following areas were identified with very low frequency: hepatobiliary disorders (n = 2), followed by immune system disorders and ear and labyrinth disorders with only 1 device each.

### 3.3. Geographical distribution

North America accounts for 40.96% (n = 104) of the total number of studies. Europe comes next with 32.28% (n = 82) of the total, then Asia (18.50%, n = 47), South America (2.36%, n = 6) and Oceania (1.97%, n = 5, all studies carried out in Australia) and Centre America (0.39%, n = 1). It is important to note that 9 studies (3.54%) did not report a specific country of origin, highlighting a limitation in the geographical traceability of DTx research efforts.

In Europe, Germany ranks as the leader in the field, accounting for 17 studies (6.69% of worldwide trials), followed by Spain (3.94%, n = 10), Italy (3.15%, n = 8), France and the United Kingdom (both at 2.36%, n = 6). A fragmented pattern was observed in other European countries, each contributing one or two studies (e.g., Austria, Greece, Poland) (Table 2).

**Table 2:** Number of clinical trials in European countries.

Region	Nr. Trials	% Trias (on worldwide trials)
**Europe**	**82**	**32,28%**
Germany	17	6,69%
Spain	10	3,94%
Italy	8	3,15%
France	6	2,36%
UK	6	2,36%
Sweden	5	1,97%
Denmark	5	1,97%
Norway	5	1,97%
Switzerland	4	1,57%
Iceland	3	1,18%
Netherlands	3	1,18%
Finland	2	0,79%
Multicountry	2	0,79%
Czechia	1	0,39%
Poland	1	0,39%
Ireland	1	0,39%
Greece	1	0,39%
Portugal	1	0,39%
Austria	1	0,39%

In Asia, significant contributions came from South Korea (5.91% of worldwide trials, n = 15) and China (6.69%, n = 17).

### 3.4. Temporal trends in DTx trials registration, status and sponsorship

The temporal distribution of the clinical trials included in this analysis provides additional insight into the evolution of the digital therapeutics (DTx) field (Fig 3). Although only a limited number of studies were registered before 2020 (8 trials in 2020), a marked increase was observed from 2021 onwards. The number of registered studies increased from 20 in 2021 to 57 in 2022 (+185%) to 59 in 2023 (+4%) and 75 in 2024 (+27%). The number of studies registered in january 2025 (n = 14) suggests that the trend is not going to reverse. Overall, these findings suggest that DTx have attracted increasing attention within clinical research, as reflected by the growing number and diversity of registered clinical trials.

**Fig 3:**
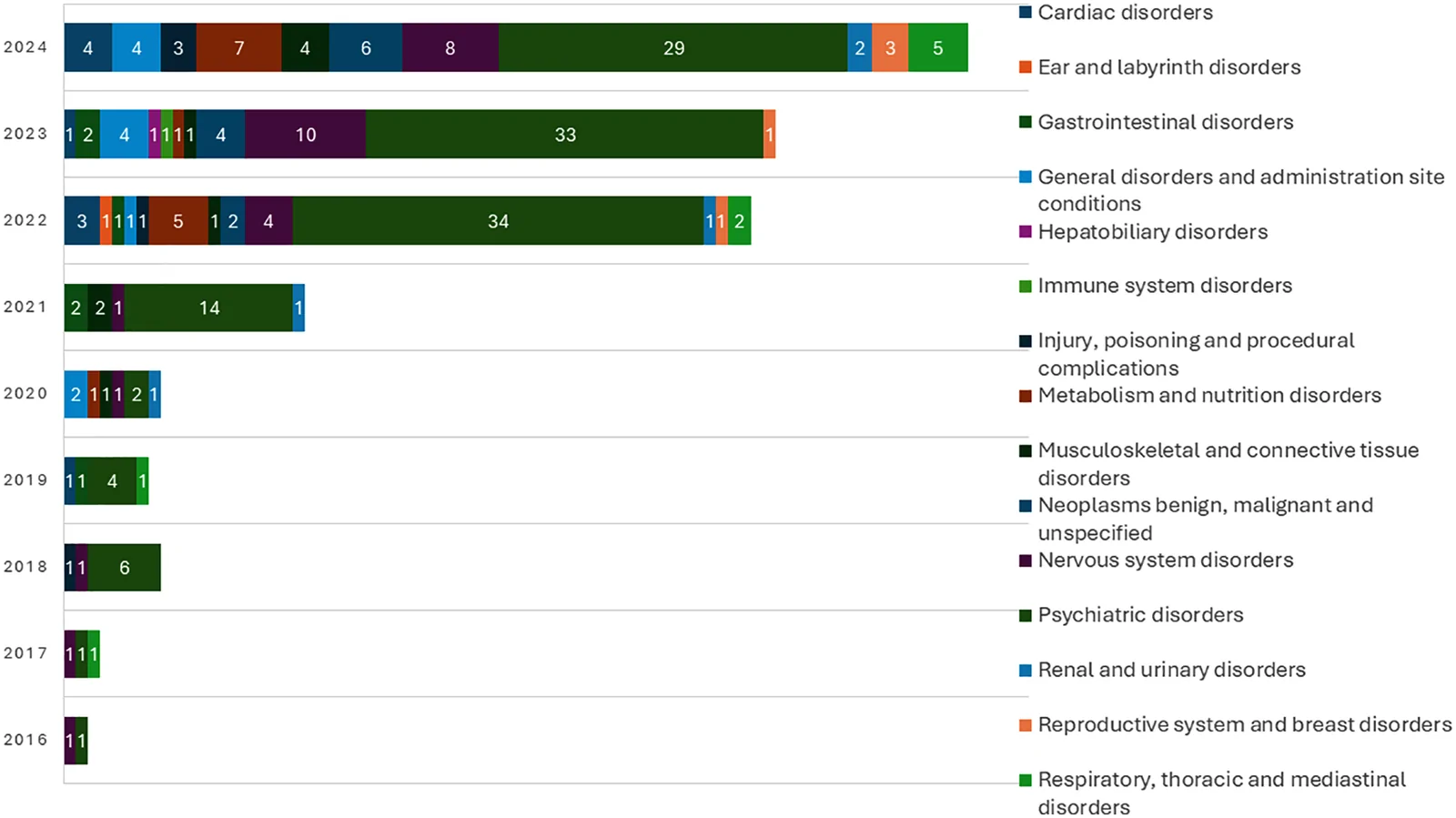
Temporal evolution of digital therapeutics clinical trials by therapeutic area (2016-2025).

The temporal evolution of therapeutic areas further illustrates how the field has developed over time. Psychiatric disorders remained the most represented therapeutic area throughout the study period. However, their share progressively decreased, from 70% of all registered studies in 2021 to 60% in 2022, 56% in 2023, and 39% in 2024. Rather than reflecting a reduction in research activity within mental health, this trend results from an increase in the number of studies conducted in other therapeutic domains. Indeed, while psychiatric applications continued to represent the largest share of registered trials, growing research activity was observed in areas such as nervous system disorders, neoplasms, metabolism and nutrition disorders, cardiac disorders, and musculoskeletal conditions. These findings suggest that, although mental health represented the primary focus of early DTx development, the field has progressively expanded towards a broader range of clinical indications, highlighting an increasing diversification of research efforts over time.

The distribution of study status further highlights the dynamic nature of the field. While 99 studies (38.98%) were completed at the time of the data extraction, a substantial proportion remained in active development, including recruiting studies (34.25%; n = 87), active but not recruiting studies (9.45%; n = 24), and not-yet-recruiting studies (16.14%; n = 41). Only a small number of studies were enrolling by invitation (1.18%; n = 3). The large proportion of ongoing studies indicates that the evidence base supporting DTx continues to expand and that numerous technologies are still undergoing clinical evaluation. Among completed studies, the mean study duration was 13.6 months, median 11.1 months and range 1.4 - 44.8 months. These findings suggest that the clinical evaluation of DTx is generally conducted over relatively short- to medium-term timeframes, although substantial variability exists across studies.

With regard to study sponsorship, most trials were funded by organizations classified as “Other” by ClinicalTrials.gov (61.02%; n = 155), a category that includes universities, hospitals, research institutions, non-profit organizations, and other sponsors not falling within industry or governmental classifications. Industry-funded studies accounted for 37.80% of the total (n = 96), whereas only a limited number of studies were funded by federal agencies (0.79%, n = 2) or other governmental organizations (0.39%, n = 1). Beyond these classifications, the analysis of sponsors and collaborators revealed frequent partnerships involving academic institutions, healthcare organizations, public research bodies, and digital health developers. Several studies were conducted through collaborations between technology companies and universities or hospitals, while others involved national research institutes and public funding agencies. Taken together, these findings suggest that DTx development is supported by a plurality of stakeholders and sponsorship models, rather than being driven by a single category of funders, suggesting that DTx development frequently involves collaborations among academic, clinical, and commercial stakeholders.

## 4. Discussion

Digital therapeutics (DTx) offer the potential to address several limitations of traditional clinical practice. They can reduce the costs associated with hospital visits or in-person consultations, enhance adherence to healthy lifestyle behaviours and prescribed treatments, enable continuous patient monitoring, and help streamline administrative tasks and routine communication [18]. DTx are inherently multifaceted, providing diverse and flexible treatment options tailored to individual needs [19] and increasing the patients’ possibilities of receiving evidence-based treatment even when conventional treatment options are limited [20]. The current body of clinical trials reflects the wide applicability of DTx and the data analysed provide insight, in particular, about the technology of the device, the therapeutic area and worldwide distribution. Correlations can also be highlighted between those variables.

From the technological point of view, mobile applications represent the more prevalent and fastest growing technology, probably due to their perceived convenience and dependence on a technological platform, the smartphone, familiar to most users. On the other hand, the necessity for developers to rely on generalist app stores is a challenge in term of patient access and safety: mechanisms have to be devised to effectively integrate DTx in the healthcare system, allowing reimbursement and access solely to the intended patients.

From the perspective of the intended use of the device, the 254 identified studies span a broad range of therapeutic areas, demonstrating that DTx can be employed either as standalone interventions or as complementary tools alongside conventional pharmacological treatments. The analysis highlights that digital therapeutics are most frequently developed to address psychiatric conditions, such as insomnia, depression, and anxiety, which together represent the dominant therapeutic focus worldwide. Cancer-related applications, particularly those aimed at improving quality of life, represent another major area of DTx in development.

The predominance of North American studies observed in the present analysis is consistent with broader evidence describing the leading role of the United States in digital investment and innovation. Recent OECD analyses have shown that digital investments have grown faster in the United States than in most OECD economies over the last decade and account for almost 90% of the difference in business investment growth between the United States and other OECD countries [21]. Furthermore, digital assets account for approximately 28% of business investment spending in the United States, compared with around 21% in the rest of the OECD. Given that Digital Therapeutics are software-based health technologies, their development is likely influenced by the same structural factors driving digital innovation more broadly. Higher levels of investment in software, databases and digital infrastructure may foster the creation, development and validation of novel digital health solutions, ultimately increasing the number of technologies entering clinical evaluation. Overall, the higher concentration of DTx clinical trials in North America may reflect the presence of a more mature digital innovation ecosystem characterised by stronger investment capacity, greater technological development and more favourable conditions for the generation of clinical evidence.

Further insight is possible when splitting the macro regions, Europe in particular, where Germany ranks as the leader, a position that can be linked to the most mature reimbursement and fast access policies. The other European Countries where reimbursement is in place, France, or where regulatory discussion has at least started, such as the UK and Italy, rank higher than the others. Other factors that may influence companies’ willingness to invest in DTx are the broader legal framework, the interoperability of DTx with the national healthcare system, the availability of funding, the digital literacy of healthcare professionals and patients’ demographics [22,23].

### 4.1. Correlation between therapeutic area and technology of the device

The analysis of the relationship between device technologies and therapeutic macro-areas (**Table B** in S1 Appendix) shows that mobile applications are the most widely adopted technology across all clinical domains examined. Psychiatric and nervous system disorders showed the greatest degree of technological diversification, including apps, web apps, videogames, virtual reality, and wearable-integrated solutions (Fig 4). This finding suggests a broader experimentation of digital intervention models in these therapeutic domains. Conversely, numerous other therapeutic areas showed limited technological diversification, relying predominantly on mobile applications (**Table B** in S1 Appendix). This distribution suggests that the degree of technological diversification varies considerably across therapeutic areas, with some clinical conditions characterized by an almost exclusive reliance on mobile applications and others presenting a broader variety of digital solutions. Furthermore, videogames and virtual reality were concentrated mainly in psychiatric and nervous system disorders, highlighting how more immersive or interactive technologies are currently being explored in a relatively limited number of therapeutic fields compared with mobile applications and web-based solutions.

**Fig 4:**
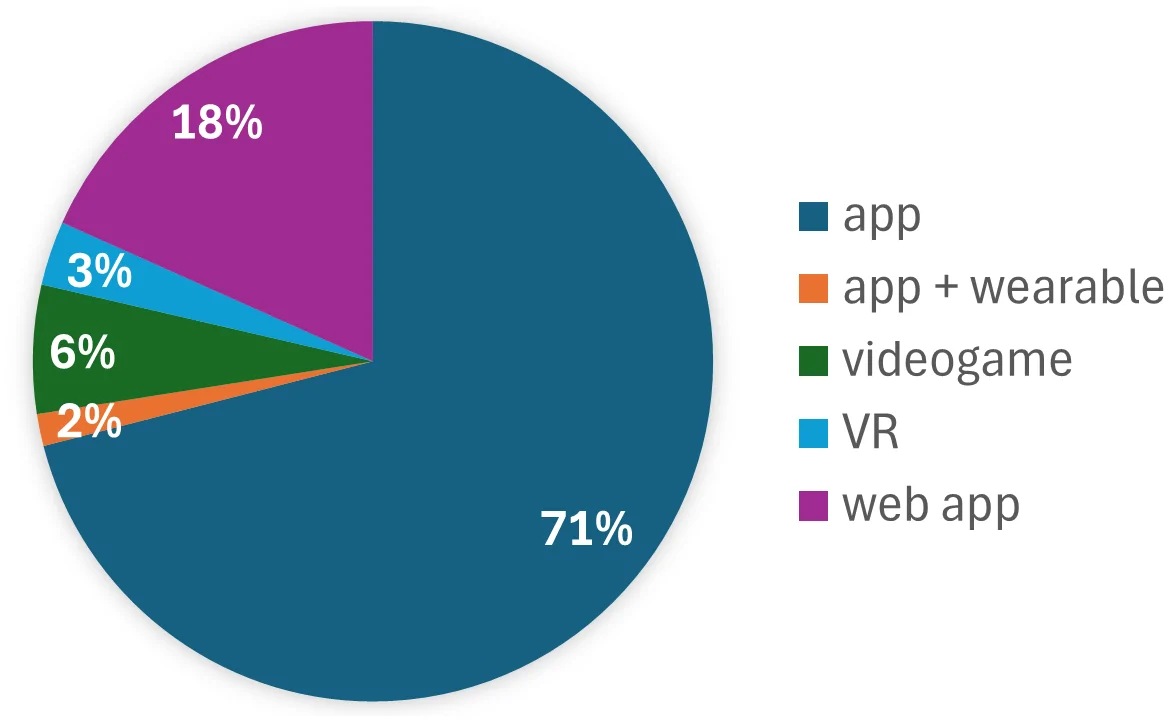
Technological devices used for psychiatric disorders (for other therapeutic areas, see Table B in S1 Appendix).

### 4.2. Correlation between therapeutic area and geographical distribution

The geographical analysis of the 254 included clinical trials reveals marked regional differences in the therapeutic areas being prioritized (**Table C** in S1 Appendix).

The majority of studies originating from America focused on psychiatric disorders (n = 76), representing 68.47% of all studies conducted in the region. In contrast, Europe displayed a more diversified therapeutic profile (Fig 5). While psychiatric disorders represent the largest category (n = 25; 30,49% of studies conducted in Europe), substantial research activity was also observed in nervous system disorders (n = 14; 17.07%), neoplasms (n = 7; 8,54%), and metabolism and nutrition disorders (n = 7; 8,54%). In Asia psychiatric disorders represent the leading area (n = 22; 46.81%) followed by nervous system disorders (n = 5; 10.64%), cardiac disorders (n = 4; 8,51%), neoplasms (n = 3; 6.38%) and metabolism and nutrition disorders (n = 2; 4.26%).

**Fig 5:**
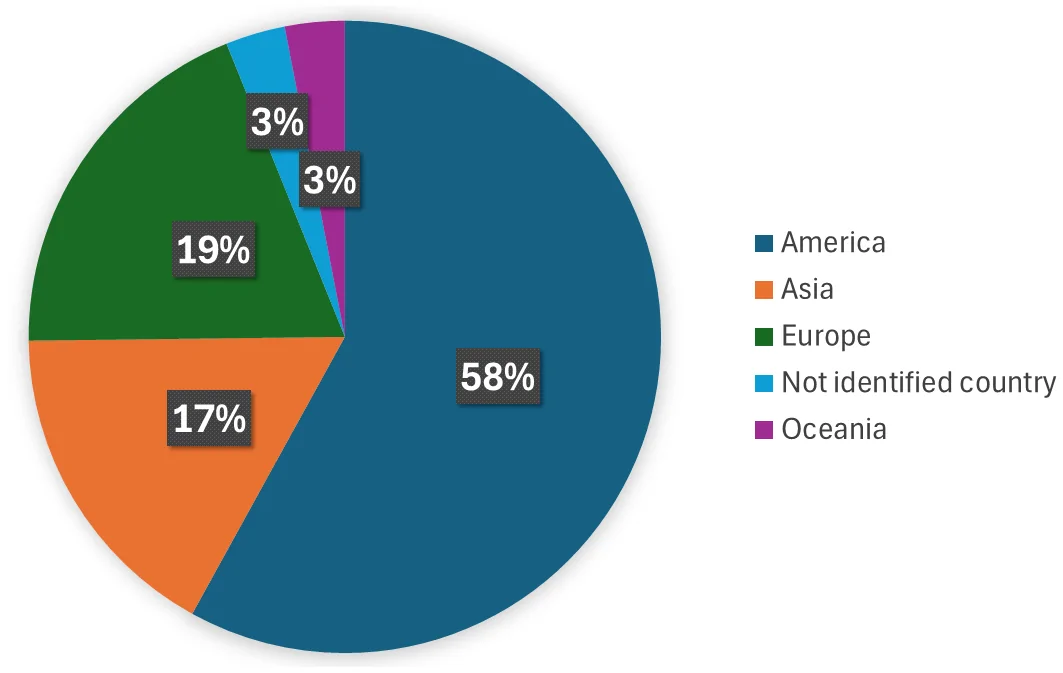
Geographical distribution of DTx clinical trials for psychiatric disorders (for other therapeutic areas, see Table B in S1 Appendix).

In general, the evolution of digital therapeutics proves to be a dynamic process, shaped by both technological advances and regional regulatory and healthcare priorities. While some areas show global convergence, such as the emphasis on mental health, others reflect distinct regional strategies and interests, suggesting the importance of contextual factors in the development and implementation of DTx solutions.

### 4.3. Challenges for the implementation of digital therapeutics

Beyond regulatory considerations, the implementation of DTx in routine clinical practice also depends on addressing important economic, ethical, and cultural challenges. From an economic perspective, the generation of clinical evidence alone is often insufficient to ensure patient access. In many healthcare systems, the absence of dedicated reimbursement pathways prevents the integration of DTx into routine care, despite demonstrated clinical benefits. Consequently, access may depend on patients’ ability to pay out-of-pocket or on local pilot initiatives, potentially generating inequalities in access to innovative treatments. Furthermore, the development and deployment of digital therapeutics are inherently complex because these interventions comprise multiple interacting and adaptive software components. Consequently, traditional evidence-generation and assessment frameworks, largely conceived for more static health technologies, may be insufficient to address the full life cycle and iterative evolution of digital therapeutics [24].

Ethical challenges associated with digital therapeutics are closely related to the governance, sharing, reuse, and protection of digital health data. The intensive processing of digital data raises concerns regarding privacy, data breaches, unlawful access, transparency, fairness, trust, secondary uses of data, and cybersecurity, all of which have been identified as key ethical issues in contemporary digital data governance frameworks [25].

Cultural and societal factors represent an additional barrier to adoption. The effectiveness of digital therapeutics depends not only on their clinical functionality but also on patients’ ability, opportunity, and motivation to engage with them. Differences in digital health literacy, age-related capabilities and attitudes toward technology, socioeconomic status, and access to internet connectivity and digital devices contribute to a digital health divide that affects uptake, sustained engagement, and ultimately health outcomes [26]. Similar challenges affect healthcare professionals, who are increasingly required to posess expertise in digital health evaluation, prescription, and monitoring to effectively integrate DTx into clinical workflows.

These considerations suggest that the successful implementation of DTx requires more than technological innovation or regulatory approval alone. Sustainable adoption will likely depend on the simultaneous development of reimbursement mechanisms, interoperable digital infrastructures, robust data governance models, and educational initiatives targeting both healthcare professionals and patients.

## 5. Conclusion

Digital therapeutics are emerging as a promising evolutionary step in healthcare, holding the potential to significantly enhance the well-being of individuals. The temporal evolution of registered clinical trials indicates a rapid expansion of DTx, accompanied by a progressive diversification of the therapeutic areas addressed, beyond mental health. However, their widespread adoption is slowed down by challenges of cultural, ethical, economic, and regulatory origin.

From the regulatory point of view, a harmonised legal definition of digital therapeutics is needed, ideally at the European level. In this context, ISO 11147 set a precedent which now holds formal significance and could serve as a starting point for national policies.

From the economic standpoint, it is crucial to provide a pathway towards reimbursement policies for DTx, and ideally for all pDMDs, and the allocation of dedicated funds.

From the ethical point of view, a balance must be struck between protecting patient privacy and making data available for research. Ensuring data interoperability through standardized languages and a federated data network is essential, allowing data to be used not only for primary purposes but also for secondary analyses.

Concerning the cultural barriers to the adoption of DTx, it’s essential to ensure that digital health technologies are well-understood by healthcare providers and the public. This includes comprehensive training for health professionals and thorough information dissemination to patients and their carers about potential benefits of digital therapeutics.

## Supporting information

S1 Appendix Additional methodological details, classification criteria, and supplementary analyses of digital therapeutics clinical trials. Methodology and source analysis: parameters and subclassification used during the analysis. Table A. Number of developed technologies by country. Table B. Type of technological device for different therapeutic areas. Table C. Therapeutic areas addressed in different geographical regions.
(DOCX)

S1 Dataset Dataset underlying the analyses reported in the manuscript. - Excel dataset containing the 254 clinical trials included in the study and the variables used for classification and analysis.
(XLSX)

## References

[1] Wang C, Lee C, Shin H. Digital therapeutics from bench to bedside. NPJ Digit Med. 2023;6(1):38. doi: 10.1038/s41746-023-00777-z 36899073 PMC10006069

[2] International Organization for Standardization. Health informatics – personalized digital health – Digital therapeutics health software systems. 2023. https://www.iso.org/standard/11147.html

[3] Regulation (EU) 2017/745 of the European Parliament and of the Council on medical devices. 2017. https://eur-lex.europa.eu/legal-content/EN/TXT/?uri=CELEX%3A02017R0745-20260719

[4] Medical Device Coordination Group. Guidance on Qualification and Classification of Software in Regulation (EU) 2017/745 – MDR and Regulation (EU) 2017/746 – IVDR (MDCG 2019-11). 2019. https://health.ec.europa.eu/document/download/b45335c5-1679-4c71-a91c-fc7a4d37f12b_en

[5] Digital Therapeutics Alliance. Understanding DTx/ DTx By Country. https://dtxalliance.org/understanding-dtx/dtx-by-country/ Accessed 2026 September 3.

[6] Minghetti P, Musazzi UM, Manellari S, Pagella V, Rocco P. Patient-managed digital medical devices: Do we need further regulation?. Informatics in Medicine Unlocked. 2024;47:101506. doi: 10.1016/j.imu.2024.101506

[7] Hong JS, Wasden C, Han DH. Introduction of digital therapeutics. Computer Methods and Programs in Biomedicine. 2021;209:106319. 10.1016/j.cmpb.2021.106319 34364181

[8] Tokarska J, Foxon G, Zakrzewska K. Evaluation and reimbursement of digital therapeutics in Germany, France, Belgium and England. In: Copenhagen, Denmark. https://www.ispor.org/docs/default-source/euro2023/isporeurope23tokarskapt21poster131258-pdf.pdf?sfvrsn=17e2dac7_0

[9] Therapeutics Alliance DTA. Is this product a DTX?. https://dtxalliance.org/wp-content/uploads/2022/09/DTA_FS_Is-This-Product-a-DTx.pdf 2022. Accessed 2026 September 3.

[10] National Library of Medicine. ClinicalTrials.gov. https://clinicaltrials.gov/ Accessed 2026 September 3.

[11] Teepe GW, Glase EM, Reips U-D. Increasing digitalization is associated with anxiety and depression: A Google Ngram analysis. PLoS One. 2023;18(4):e0284091. doi: 10.1371/journal.pone.0284091 37027368 PMC10081798

[12] Bantjes J. Digital solutions to promote adolescent mental health: Opportunities and challenges for research and practice. PLoS Med. 2022;19(5):e1004008. doi: 10.1371/journal.pmed.1004008 35639740 PMC9154099

[13] Sander LB, Beisemann M, Karyotaki E, van Ballegooijen W, Cuijpers P, Teismann T, et al. Effects of digital cognitive behavioral therapy for depression on suicidal thoughts and behavior: Protocol for a systematic review and meta-analysis of individual participant data. PLoS One. 2023;18(6):e0285622. doi: 10.1371/journal.pone.0285622 37289758 PMC10249902

[14] Koller C, Blanchard M, Hügle T. Assessment of digital therapeutics in decentralized clinical trials: A scoping review. PLOS Digit Health. 2025;4(6):e0000905. doi: 10.1371/journal.pdig.0000905 40549731 PMC12185025

[15] Choi-Kain LW, Murray GE, Jurist J, Ren B, Germine L. Online psychoeducation and digital assessments as a first step of treatment for borderline personality disorder: A protocol for a pilot randomized controlled trial. PLoS One. 2023;18(12):e0294331. doi: 10.1371/journal.pone.0294331 38060545 PMC10703320

[16] Aapro M, Bossi P, Dasari A, Fallowfield L, Gascón P, Geller M, et al. Digital health for optimal supportive care in oncology: benefits, limits, and future perspectives. Support Care Cancer. 2020;28(10):4589–612. doi: 10.1007/s00520-020-05539-1 32533435 PMC7447627

[17] Ellahham S. Artificial Intelligence: The Future for Diabetes Care. Am J Med. 2020;133(8):895–900. doi: 10.1016/j.amjmed.2020.03.033 32325045

[18] Abbadessa G, Brigo F, Clerico M, De Mercanti S, Trojsi F, Tedeschi G, et al. Digital therapeutics in neurology. J Neurol. 2022;269(3):1209–24. doi: 10.1007/s00415-021-10608-4 34018047 PMC8136262

[19] Phan P, Mitragotri S, Zhao Z. Digital therapeutics in the clinic. Bioeng Transl Med. 2023;8(4):e10536. doi: 10.1002/btm2.10536 37476062 PMC10354777

[20] Niiberg-Pikksööt T, Laas K, Aluoja A, Braschinsky M. Implementing a digital solution for patients with migraine-Developing a methodology for comparing digitally delivered treatment with conventional treatment: A study protocol. PLOS Digit Health. 2024;3(2):e0000295. doi: 10.1371/journal.pdig.0000295 38421955 PMC10903846

[21] Gal P, Hooley J, Ozturk F, Unsal F. Business investment in the face of the digital transformation: Initial evidence, OECD Economics Department Working Papers. 2026. Paris: OECD Publishing. doi: 10.1787/4f89aa3e-en

[22] van Kessel R, Roman-Urrestarazu A, Anderson M, Kyriopoulos I, Field S, Monti G, et al. Mapping Factors That Affect the Uptake of Digital Therapeutics Within Health Systems: Scoping Review. J Med Internet Res. 2023;25:e48000. doi: 10.2196/48000 37490322 PMC10410406

[23] Dahlhausen F, Zinner M, Bieske L, Ehlers JP, Boehme P, Fehring L. There’s an app for that, but nobody’s using it: Insights on improving patient access and adherence to digital therapeutics in Germany. Digit Health. 2022;8:20552076221104672. doi: 10.1177/20552076221104672 35811758 PMC9260569

[24] Kim M, Patrick K, Nebeker C, Godino J, Stein S, Klasnja P, et al. The Digital Therapeutics Real-World Evidence Framework: An Approach for Guiding Evidence-Based Digital Therapeutics Design, Development, Testing, and Monitoring. J Med Internet Res. 2024;26:e49208. doi: 10.2196/49208 38441954 PMC10951831

[25] Eke D, Stahl B. Ethics in the governance of data and digital technology: An analysis of European data regulations and policies. AI and Ethics. 2024. doi: 10.1007/s44206-024-00101-6

[26] Western MJ, Smit ES, Gültzow T, Neter E, Sniehotta FF, Malkowski OS, et al. Bridging the digital health divide: a narrative review of the causes, implications, and solutions for digital health inequalities. Health Psychol Behav Med. 2025;13(1):2493139. doi: 10.1080/21642850.2025.2493139 40276490 PMC12020140

