# Supplementary material for: Pathologies and technologies for digital therapeutics: An analysis of global clinical trials
Source: PLOS Digit Health. 2026 Oct 14;5(10):e0001750. doi: 10.1371/journal.pdig.0001750 (PMC13667809; doi:10.1371/journal.pdig.0001750)
Supplement: Supplementary file 1 [file pdig.0001750.s001.docx]

**S1 Appendix**

1. **Methodology and source analysis**

During the analysis, the following parameters and related subclassifications were investigated:

| **Country** | |
| --- | --- |
| **America** | Central America |
|  | North America |
|  | South America |
| **Asia** | China |
|  | Israel |
|  | Korea |
|  | Pakistan |
|  | Russian federation |
|  | Taiwan |
|  | Turkey |
| **Europe** | Austria |
|  | Czechia |
|  | Denmark |
|  | Finlandia |
|  | France |
|  | Germany |
|  | Greece |
|  | Iceland |
|  | Ireland |
|  | Italy |
|  | Netherlands |
|  | Norway |
|  | Poland |
|  | Portugal |
|  | Spain |
|  | Sweden |
|  | Switzerland |
|  | UK |
|  | Multi-country |
| **Oceania** | Australia |
| **N/A** | Country not identified in study |

**Technology**

Application (app)

Web application (web app)

Virtual Reality (VR)

Videogame

App + wearable

**Therapeutic area (MedDra, SOC)**

Psychiatric disorders

Nervous system disorders

Neoplasms benign, malignant and unspecified

Metabolism and nutrition disorders

General disorders and administration site conditions

Cardiac disorders

Musculoskeletal and connective tissue disorders

Respiratity, thoracic and mediastinal disorders

Renal and urinary disorders

Gastrointestinal disorders

Reproductive system and breast disorders

Injury, poisoning and procedural complications

Hepatobiliary disorders

Immune system disorders

Ear and labyrinth disorders

2. **Supplementary tables**

**Table A. Number developed technologies by Country**

| **Region/Country** | **app** | **app + wearable** | **videogame** | **VR** | **web app** | **Total** |
| --- | --- | --- | --- | --- | --- | --- |
| **America** | **80** | **3** | **6** | **4** | **18** | **111** |
| Centre America | 1 | - |  | - | - | 1 |
| North America | 74 | 3 | 6 | 4 | 17 | 104 |
| South America | 5 | - | - | - | 1 | 6 |
| **Asia** | **27** | **2** | **6** | **5** | **7** | **47** |
| China | 9 | 1 | 3 |  | 4 | 17 |
| Israel | 3 | - | - | - | - | 3 |
| Pakistan | 2 | - | - | 2 | - | 4 |
| Russian federation | - | - | - | 1 | - | 1 |
| Taiwan | 1 | - | 1 | 1 | - | 3 |
| Turkey | 1 | - | 2 | - | 1 | 4 |
| South Korea | 11 | 1 |  | 1 | 2 | 15 |
| **Europe** | **46** | **2** | **1** | **8** | **25** | **82** |
| Austria | 1 | - | - | - | - | 1 |
| Czechia | 1 | - | - | - | - | 1 |
| Denmark | 3 | - | - | - | 2 | 5 |
| France | 4 | - | - | 2 | - | 6 |
| Germany | 6 | 1 |  |  | 10 | 17 |
| Greece | - | - | - | - | 1 | 1 |
| Iceland | 2 | - | - | - | 1 | 3 |
| Ireland | 1 | - | - | - | - | 1 |
| Italy | 4 | 1 | - | 2 | 1 | 8 |
| Multicountry | 2 | - | - | - | - | 2 |
| Netherlands | 2 | - | - | 1 | - | 3 |
| Norway | 2 | - | - | - | 3 | 5 |
| Poland | 1 | - | - | - | - | 1 |
| Portugal | - | - | - | - | 1 | 1 |
| Spain | 6 | - | - | 1 | 3 | 10 |
| Sweden | 3 | - | - | - | 2 | 5 |
| Switzerland | 3 | - | - | - | 1 | 4 |
| UK | 5 | - | - | 1 | - | 6 |
| Finland | - | - | 1 | 1 | - | 2 |
| **Not identified country** | **8** |  |  |  | **1** | **9** |
| Not identified country | 8 | - | - | - | 1 | 9 |
| **Oceania** | **5** |  |  |  |  | **5** |
| Australia | 5 | - | - | - | - | 5 |
| **Total** | **166** | **7** | **13** | **17** | **51** | **254** |

**Table B. Type of technological device for different therapeutic areas**

| **Therapeutic area** | **app** | **app + wearable** | **videogame** | **VR** | **web app** | **Total** |
| --- | --- | --- | --- | --- | --- | --- |
| Psychiatric disorders | 93 | 2 | 8 | 4 | 24 | 131 |
| Nervous system disorders | 13 | 3 | 3 | 7 | 2 | 28 |
| Neoplasms benign, malignant and unspecified | 9 | - | - | - | 5 | 14 |
| Metabolism and nutrition disorders | 10 | - | - | - | 4 | 14 |
| General disorders and administration site conditions | 6 | - | - | 3 | 2 | 11 |
| Cardiac disorders | 6 | 1 | - | 1 | 3 | 11 |
| Musculoskeletal and connective tissue disorders | 6 | 1 | - | 1 | 2 | 10 |
| Respiratory, thoracic and mediastinal disorders | 4 | - | 1 | 1 | 3 | 9 |
| Renal and urinary disorders | 6 | - | - | - | - | 6 |
| Gastrointestinal disorders | 5 | - | - | - | 1 | 6 |
| Reproductive system and breast disorders | 3 | - | - | - | 2 | 5 |
| Injury, poisoning and procedural complications | 2 | - | 1 | - | 2 | 5 |
| Hepatobiliary disorders | 2 | - | - | - | - | 2 |
| Ear and labyrinth disorders | 1 | - | - | - | - | 1 |
| Immune system disorders | - | - | - | - | 1 | 1 |
| **Total** | **166** | **7** | **13** | **17** | **51** | **254** |

**Table C. Therapeutic areas addressed in different countries**

| **Therapeutic area** | **America** | **Asia** | **Europe** | **Not identified country** | **Oceania** | **Total** |
| --- | --- | --- | --- | --- | --- | --- |
| Psychiatric disorders | 76 | 22 | 25 | 4 | 4 | 131 |
| Nervous system disorders | 9 | 5 | 14 | - | - | 28 |
| Metabolism and nutrition disorders | 3 | 2 | 7 | 2 | - | 14 |
| Neoplasms benign, malignant and unspecified | 4 | 3 | 7 | - | - | 14 |
| Cardiac disorders | 1 | 4 | 5 | 1 | - | 11 |
| General disorders and administration site conditions | 5 | 1 | 5 | - | - | 11 |
| Musculoskeletal and connective tissue disorders | 4 | 3 | 3 | - | - | 10 |
| Respiratory, thoracic and mediastinal disorders | 2 | 3 | 3 | - | 1 | 9 |
| Gastrointestinal disorders | 4 | 1 | 1 | - | - | 6 |
| Renal and urinary disorders | 1 | 1 | 4 | - | - | 6 |
| Injury, poisoning and procedural complications | 1 | 1 | 3 | - | - | 5 |
| Reproductive system and breast disorders | - | 1 | 3 | 1 | - | 5 |
| Hepatobiliary disorders | - | - | 1 | 1 | - | 2 |
| Ear and labyrinth disorders | 1 | - | - | - | - | 1 |
| Immune system disorders | - | - | 1 | - | - | 1 |
| **Total** | **111** | **47** | **82** | **9** | **5** | **254** |
